# The ultrastructure of nymphal dermal pores and the genitalia of adult male of *Phenacoccus solenopsis* (Hemiptera: Pseudococcidae)

**DOI:** 10.1038/s41598-024-51233-1

**Published:** 2024-01-12

**Authors:** Nesreen M. Abd El-Ghany, Shadia E. Abd El-Aziz, Michel J. Faucheux

**Affiliations:** 1 Pests and Plant Protection Department, Agricultural and Biological Research Institute, National Research Centre, 33 El-Buhouth Street (Formerly El-Tahrir St.), Dokki, P.O. Box 12622, Giza, Egypt; 2 Laboratoire d’Endocrinologie des Insectes Sociaux, Université de Nantes, 2 Rue de la Houssinière, B. P. 92208, 44322 Nantes Cedex 3, France

**Keywords:** Entomology, Zoology, Biological techniques, Microscopy, Scanning probe microscopy

## Abstract

The cotton mealybug, *Phenacoccus solenopsis*, has established itself as an invasive insect pest worldwide. It causes structural and physiological damage to various crops and can cause substantial financial losses in their production. The successful reproduction of this pest under a wide range of conditions is a key to its success. Despite this, the morphology of its genitalia, genital sensilla, and wax-producing dermal pores has received little attention, with little descriptions of their ultrastructure. By investigating those features with SEM, the present study revealed considerable new insights into the identification of the nymphal and adult stages of *P. solenopsis*. In addition, the description of the ultrastructural genital morphology of the immature stages of *P. solenopsis* has revealed characteristics that facilitate their discrimination. Trilocular pores were observed on both sides of the body, while the quinquelocular pores were distributed only on the ventral surface in both the first and second nymphal instars. The adult male is characterized by two pairs of waxy caudal filaments surrounded by clusters of 55 to 60 stellate pores, and each pregenital segment bears a pair of stellate pores composed of 4 or 5 peripheral loculi. Sensilla trichodea and numerous microtrichia are present on the pregenital segments. The penile sheath bears three subtypes of sensilla basiconica and also campaniformia, whereas the style bears three subtypes of sensilla campaniformia. The findings of this study could assist in the identification of the adult and nymphal stages of *P. solenopsis*, and also provide insights into the structures found on the genitalia of the adult male that possibly have an important role in mating events and copulatory behavior. Furthermore, these findings were able to contribute to better understanding the functional morphology of *P. solenopsis*.

## Introduction

Scale insects, which are distributed worldwide, are classified into 22 families including Pseudococcidae (mealybugs)^1^. Mealybugs are distinguished by their production of fine, powdery, integumentary wax. The hydrophobic layers of the wax are arranged in long, lateral filaments, hence the common name, “mealybug”, attributed to the Pseudococcidae^2^. The integument is an important protective tissue that prevents inner moisture from excessive evaporation and environmental attacks by inorganic compounds, pathogens, microorganisms, and insecticide sprays^3^. Gullan and Cranston suggested that the wax helps protect the mealybug from predators and insecticides across the different developmental stages of its life cycle^3^. The types and distribution of pores and ducts associated with wax production have been considered by taxonomists as invaluable characters for the identification and classification of mealybugs and other scale insects^4–10^. On the basis of the dermal pores producing the wax, Cox and Pearce distinguished three species of mealybugs, *Ferrisia virgate* (Cockerell), *Phenacoccus manihoti* (Matile-Ferrero), and *Planococcus citri* (Risso)^11^.

In Pseudococcidae, the males go through six developmental stages, namely the egg, first instar nymph, second instar nymph, prepupa, pupa, and adult. The male is usually slim in appearance and easily distinguishable from the adult females. Adult males resemble aphids in that they have a non-functional mouth, 9–10 segmented antennae, a pair of tiny mesothoracic wings, metathoracic hamulohalterae, 5-segmented legs, an abdomen that bears 0–3 pairs of caudal filaments associated with tail-forming pore clusters, and an aedeagus, distinguished by a penile sheath, at the end of the abdominal segments^1^.

The cotton mealybug, *Phenacoccus solenopsis* Tinsley, has become established as an invasive pest in the Afrotropical, Australasian, Nearctic, Neotropical, and Oriental regions, and causes direct economic and ecological damage to crops and native flora^12^. The adult females weigh about 100 to 200 times more than the adult males^13^. Mealybug females are neotenic and have three nymphal instars; in contrast, the males go through two nymphal instars and additional prepupal and pupal stages, which develop inside a waxy cocoon^14,15^. Females live for several months, whereas the tiny, winged male is ephemeral (2–3 days). The adult male remains inside the wax webbing of the pupal cocoon for 4–12 h before emerging. It resembles a fluffy gnat and has two pairs of waxy caudal filaments, whereas most mealybug species have one pair of waxy caudal filaments. The wax tail consists of two pairs of caudal filaments that are secreted by clusters of glandular pouch pleural pores located on the 7th and 8th abdominal segments^16–18^. The length of these filaments increases from the time of the emergence of the adult male until maturation occurs. The adult male takes 1–2 days to complete the formation of its wax tail, which assists in flight stabilization^19^.

The mature male is polygynous in that it mates with different females during its lifetime. Seven mealybug species of the genera, *Pseudococcus, Planococcus,* and *Nipaecoccus*, were studied to estimate at which physiological age the males are sexually active and for how long^20,21^. They determined that the adult males take 30–40 h to achieve sexual maturity and being able to fly. The lengths of the caudal filaments ranging from 0.96 to 1.15 mm for seven mealybug species which are the spherical mealybug, *Nipaecoccus viridis* Newstead; the cypress mealybug, *Planococcus vovae* Nasonov; the citrus mealybug, *Pl. citri Risso*; the vine mealybug, *Pl. ficus* Signoret; the citriculus or cryptic mealybug, *Pseudococcus cryptus* Hempel; the long-tailed mealybug, *Ps. longispinus* Targioni Tozzetti; and the obscure mealybug, *Ps. viburni* Signoret^20^.

Identification of the various species of mealybug is usually based on the morphological features of the specimens^22^. Easy separation of the adult female from the nymphal stages for all species is based on their characteristic genital structure called the vulva, present posteriorly on the ventral side of the female abdomen and absent in the nymphal stages. However, differentiation in such cases takes a long time, approximately 1 month, to enable the adult female to be distinguished. Therefore, rapid and accurate identification is in demand and is a critical step for both the control of the pests and for agricultural quarantine purposes regarding imported and exported crops. Furthermore, identifying the immature stages of mealybugs allows accelerated decision-making for control strategies.

Previous studies have provided important morphological descriptions of the adult male of *P. solenopsis*^11,22^, but the genitalia and structures associated with wax production, such as the dermal pores and ducts, have received little attention. Moreover, descriptions of the ultrastructural features of the adult male genitalia, the types and distribution of sensilla, and the dermal pores, are limited.

For the adult male of *P. solenopsis*, few studies have been performed on the pleural pores of the glandular pouch as a form of wax pore different from those of the other immature stages, although, the structure of the pleural pores and ducts of four species from 4 genera of Pseudococcidae, namely *Ferrisia virgate* Cockerell*, P. manihoti* Matile-Ferrero, *Pl. citri* Risso, *Maconellicoccus hirsutus* Green)^5,11^, have been described. These articles only contain scanning electron micrographs of the glandular pouch, pleural pores, and ducts.

For this reason, the present study aimed to contribute to the identification of the nymphal and adult stages of the *P. solenopsis* male by describing the ultrastructural morphology of their dermal pores. Moreover, the study explores the morphometry of the genitalia and its sensilla types. The importance of such knowledge is that it would provide an identification key for the rapid diagnosis of the nymphal stages found on crops at pre-export inspections. The present study provides insights into the structures found on the genitalia of the adult male *P. solenopsis* that possibly having an important role in mating events and copulatory behavior.

## Results

### Immature stages

#### Morphology of the nymphal instars

**Figure 1:**
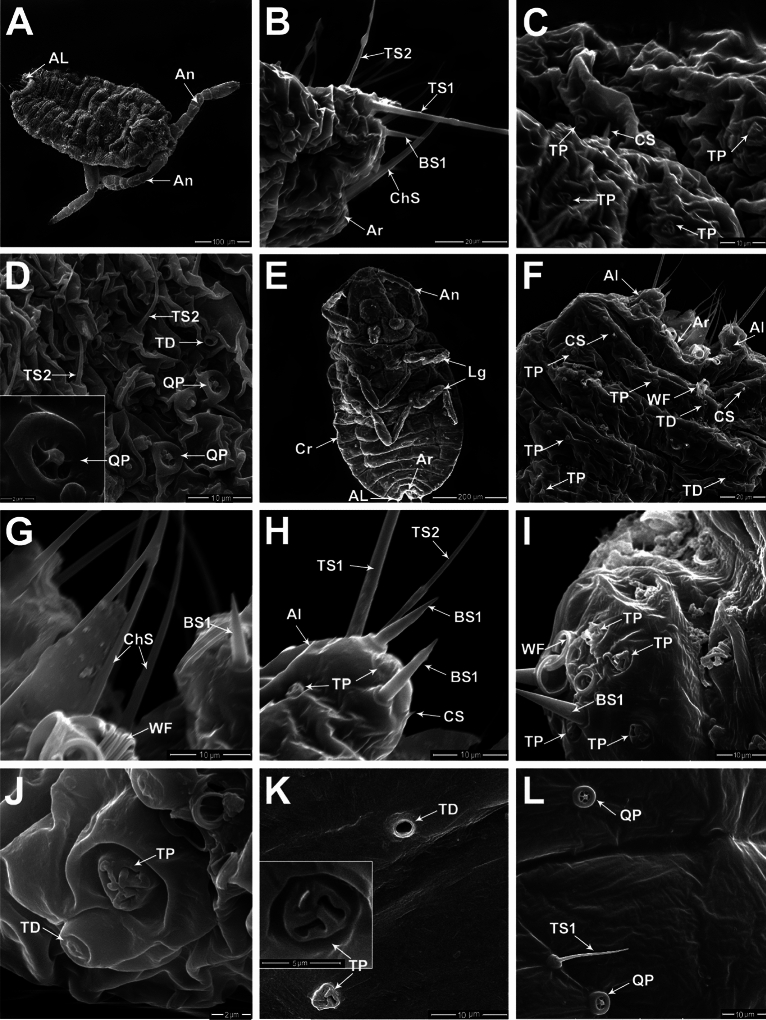
Nymphal instars of *P. solenopsis* male: (**A–D**) Showing different sensillum types distributed on the first instar nymph. (**A**) Dorsal view of the first instar nymph, showing the whole body with 7-segmented antennae (An) and pair of anal lobe (AL) at the distal end. (**B**) Magnified lateral view of the anal lobe cerarius (AL) with sensillum basiconicum (BS1); two types of sensilla trichodea (TS1, TS2), and a part of the anal ring (Ar) associated with sensilla chaetica (ChS). (**C**) Dorsal view of the integument of the first instar nymph with various groups of trilocular pores (TP) and short sensillum conical (CS). (**D**) Ventral view of the 1st instar nymph shows various sensilla trichodea (TS2), quinquelocular pores (QP), and tubular ducts (TD). Magnified view of quinquelocular pore (QP) at the bottom left of the micrograph. (**E–L**) The second instar nymph; (**E**) Ventral view of complete second instar nymph (An: Antenna, Lg: Leg, Ar: Anal ring, Al: Anal lobe, Cr: cerari), (**F**) Magnified view of the last abdominal segments showing various groups of trilocular pores (TP); wax filaments (WF), short sensillum conical (CS) distributed along the integument; anal lobes (AL), and anal ring (Ar) in the medial area. (**G**) Magnified view of the anal ring with associated sensilla chaetica (ChS); group of wax filaments (WF), and sensillum basiconicum (BS1) on the anal lobe. (**H**) Magnified view of the anal lobe cerarius with associated pair of sensilla basiconica (BS1), two types of sensilla trichodea (TS1, TS2), trilocular pores (TP), and short sensillum conical (CS). (**I**) Lateral view of the anal lobe cerarius, showing a pair of sensilla basiconica (BS1); trilocular pores (TP) associated with wax filaments (WF); (**J**) Magnified view of trilocular pores (TP) and tubular ducts (TD) on the dorsal surface of the second instar nymph. (**K**) Magnified view of trilocular pores (TP) and tubular ducts (TD) on the ventral side of the second instar nymph. (**L**) Ventral view of the second instar nymph associated with quinquelocular pores (QP) and short sensilla trichodea (TS2).

The first instar nymph (crawler) of *P. solenopsis* is mobile, with well-developed, 5-segmented legs, 6-segmented antennae, 18 pairs of cerari, trilocular wax pores, and tubular ducts. The total length of the first instar nymph is 322.54 ± 13.34 µm and the width is about half the value of its length at 150.05 ± 5.37 µm (Fig. 1A). The second instar nymph is more pronounced than the first instar nymph, with a total length ranging from 0.63 to 0.66 mm. The second instar nymph of the male appeared more elliptical than the more ovoid females. Nymphal stage is characterized by 7-segmented antennae (Fig. 1E). A thin powdery secretion covers most of the body dorsum. The body margin has 18 pairs of cerarii, with each cerarius consisting of two small conical sensilla (CS, length 2.63 ± 0.05 µm) that are surrounded by groups of trilocular pores throughout the body surface (Fig. 1F).

#### Types of wax pores and sensilla of the nymphal instars

In the first instar nymph, an aggregation of auxiliary sensilla (two types of sensilla trichodea, TS1 & TS2), sensillum basiconicum (BS1) and lanceolate setae, and 1–2 trilocular pores, are associated with the anal lobe (Fig. 1B). TS1 is a hair-like structure that is thicker at the base and tapers towards the tip. The TS2 is a short to medium length hair-like structure, with straight or slightly curved with a slightly sharp tip. The mean lengths of TS1 and TS2 are 69.80 ± 7.08 µm and 32.20 ± 3.43 µm, while their width measures 2.69 ± 0.13 µm and 1.06 ± 0.05 µm, respectively.

Two types of wax pores, trilocular (TP) and quinquelocular (QP), are present on the integument of nymphal instars. Trilocular pores were detected on both sides (dorsal and ventral), being more abundant on the dorsal surface (Fig. 1C). The TP consists of a triangular depression along each edge with three elongated, dumbbell-shaped loculi (Fig. 1C). In contrast, the quinquelocular pores were located only on the ventral surface of the body (Fig. 1D). In addition, tubular ducts (TD) were observed on both the dorsal and ventral surfaces.

For the 2nd instar nymph, the anal ring (39–44 µm in width) has associated with 6 long sensilla chaetica (ChS). ChS is characterized by a slightly grooved wall and sharp tip (35.46 ± 1.78 µm in length; 1.69 ± 0.14 µm in basal diameter), located between the two anal lobe cerari (Fig. 1F,G). A cerarius consists of an aggregation of auxiliary sensilla (also called “auxiliary setae”) composed of two subtypes of sensilla trichodea (TS1 & TS2), a pair of sensilla basiconica (BS1), and 4–5 trilocular pores (Fig. 1H,I). TS1 and TS2 are characterized by a smooth wall hair-like structures with sharp tips with varied lengths of 57.33 ± 2.50 µm and 29.0 ± 0.82 µm, respectively. The BS1 is a smooth-walled sensillum with a mean length of 10.22 ± 0.39 µm and 1.80 ± 0.08 µm in diameter. Various trilocular pores are distributed throughout both surfaces of the body. They produce curled wax filaments which resemble a railway track in shape, ranging from 1.30 to 1.55 µm (Fig. 1G,I). The TPs consisted of a triangular depression with three elongated, dumbbell-shaped loculi (1.70 ± 0.06 µm length of the opening of the loculi) (Fig. 1J,K).

Tubular ducts (TD) were observed on both the dorsal and ventral surfaces in the two nymphal instars of the mealybug male. These TDs are more numerous and abundance in the second instar male, which appeared as circular apertures with a slightly elevated rim of 1.46 ± 0.15 µm diameter; uniloculus (Fig. 1J,K). Similar to the first instar nymph, QPs were distributed only on the ventral surface of the second instar nymph, in the middle parts of the segments (Fig. 1L).

#### Prepupal and pupal stages

**Figure 2:**
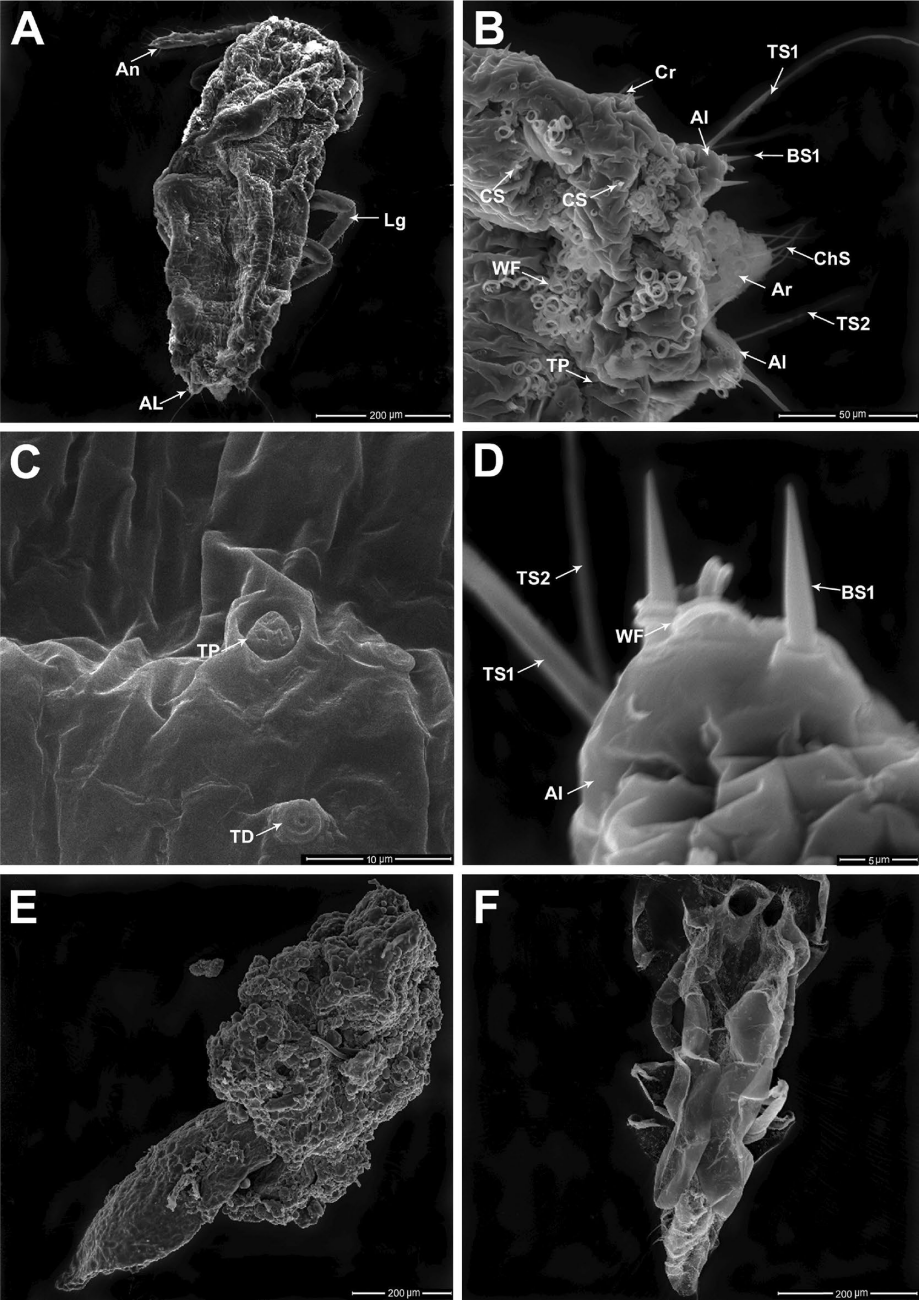
Pupation of *P. solenopsis* male: (**A**) Lateral view of prepupal stage characterized by 7-segmented antenna (An), 3 pairs of legs (Lg), a pair of anal lobes (AL) at the distal end, and excess of secreted wax filaments. (**B**) Magnified view of the last two abdominal segments showing various groups of wax filaments (WF) secreted from trilocular pores (TP), short sensillum conical (CS), Cr: cerari, anal ring (Ar) in the medial area bears 6 sensilla chaetica (ChS), anal lobe with a pair of sensilla basiconica (BS1), sensilla trichodea (TS1, TS2). (**C**) Magnified view of trilocular pore (TP) and tubular duct (TD). (**D**) Magnified view of the anal lobe cerarius of the prepupa with a pair of sensilla basiconica (BS1), sensillum trichodeum (TS1), sensillum trichodeum (TS2), and folded wax filaments (WF) secreted from trilocular pore. (**E**) Pupal stage covered by layers of wax filaments. (**F**) The molted skin after male adult emergence.

Pupation of the *P. solenopsis* male includes two instars, the prepupal and pupal stages, each of which occurs inside a fluffy puparium composed of waxy threads. The prepupal stage becomes more elliptical than the second instar nymph and has a total length of 1.05–1.08 mm (Fig. 2A). It is characterized by numerous tubular ducts, particularly on the ventral surface of the abdomen. Protruding from the anal ring, a pipe of densely matted, fine wax filaments is supported by long sensilla chaetica with a mean length of 45.05 ± 1.22 µm and 1.60 ± 0.08 µm in diameter (Fig. 2B). Trilocular pores and TDs are distributed throughout the integument (Fig. 2C). The anal lobe is associated with a single long TS1 (91.04 ± 1.73 µm in length; 1.86 ± 0.05 µm in diameter), and a single thin TS2 (39.67 ± 3.17 µm in length; 0.67 ± 0.06 µm in diameter), and a pair of BS1 (12.36 ± 1.15 µm in length; 1.83 ± 0.13 µm in diameter).

There are also trilocular pores which produce curled, railway track-shaped wax filaments (1.40 to 1.45 µm in width) (Fig. 2D). In the pupal stage, the TDs are mainly produced several longitudinal waxed ridges that are sticky and known to be utilized in forming its cocoon (Fig. 2E). The full length of pupae ranging from 1.45 to 1.61 mm. Ecdysis of the emerged adult male, seen as the shedding of the pupal cuticle, reveals an elliptical body that has a length of 1.3 mm and a width of 0.3 mm (Fig. 2F).

### Adult male

**Figure 3:**
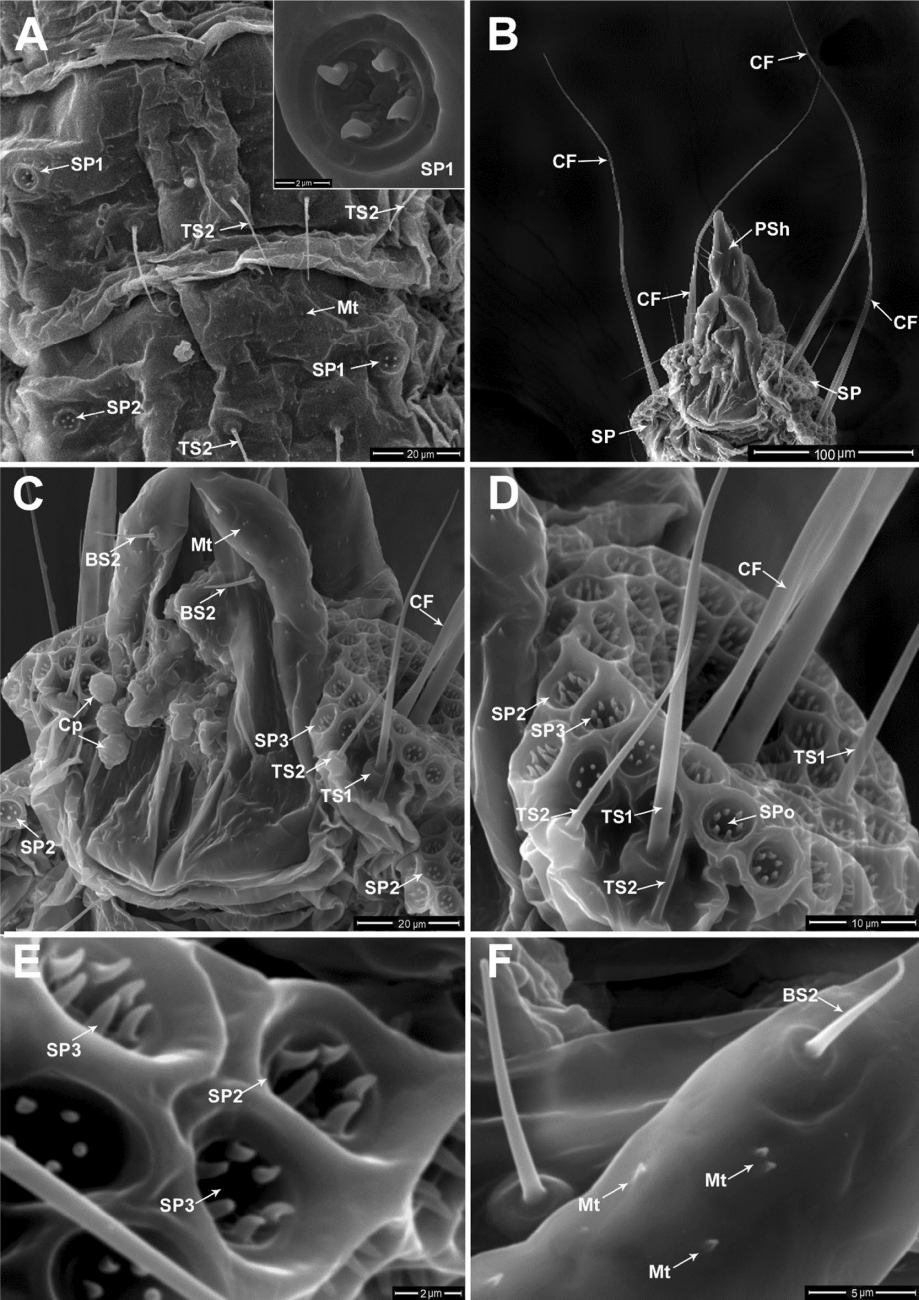
Wax pores associated with adult male of *P. solenopsis*: (**A**) The 5th and 6th abdominal segments bear sensilla trichodea (TS2), each segment laterally bears a pair of stellate pores (SP1) or (SP2), rows of microtrichia (Mt) are distributed on the integument; at the top-right of the micrograph, magnified view of stellate pore (SP1) with 4 peripheral loculi resembling short sensillum basiconicum. (**B**) Ventral view of the 7th–10th abdominal segments, with the genital segments (9th and 10th) fused and forming the penile sheath (PSh) and two pairs of caudal filaments (CF) surrounded by various groups of stellate pores (SP) on the lateral surface of the 7th and 8th abdominal segments. (**C**) Magnified view of the last three abdominal segments showing two types of stellate pores (SP2, SP3), a chaplet of 3 spores (Cp), two types of sensilla trichodea (TS1, TS2), a pair of sensilla basiconica (BS2), and microtrichia (Mt) on the medial part of 9th abdominal segment. (**D**) Magnified view of the stellate pores (SP2, SP3) with central opening (SPo) surrounding a pair of caudal filaments (CF) at the center and two types of sensilla trichodea (TS1, TS2). (**E**) Magnified view of stellate pores; SP2 associated with 5 peripheral loculi resembling short sensillum basiconicum: SP3 associated with 6 peripheral loculi. (**F**) Magnified view of the sensillum basiconicum (BS2) and microtrichia (Mt) distributed on the penile sheath.

The adult male of *P. solenopsis* is characterized by a slender and narrow body with a pair of well-developed forewings and modified hindwings in a hamulohalterae. It is distinguished by a largely membranous abdomen, becoming gradually narrower posteriorly. The abdomen is composed of 10 segments; the eight pregenital segments are distinct, but the genital segments (9th and 10th) are fused. The adult male is also distinguished by the presence of various glands and ducts, which are usually absent or reduced in the females and immature stages (Fig. 3).

Each pregenital segment bears a pair of lateral stellate pores (SP1 and SP2) which secrete wax that covers the male body, SP1 has 4 projections (peripheral loculi) resembling short sensillum basiconicum. Sensilla trichodea subtype 2 and numerous microtrichia were observed on each pregenital segment (Fig. 3A). The adult male also displays two pairs of waxy caudal filaments (CF), with mean lengths of 245.73 ± 12.64 µm, which are located on the 7th and 8th abdominal segments (Fig. 3B). The ratio of the caudal filament length to the total body length was1:4.64. The caudal filaments arise from the center of the tail-forming pore, which is known as the glandular pouch. The pouch is composed of a cluster of stellate pores on the lateral surfaces of the seventh and eighth abdominal segments (Fig. 3B–D). The caudal filament is set in a small, cup-shaped depression surrounded by a cluster of 55–60 stellate pores (SP2 and SP3), which secrete the wax that covers the caudal filaments. Each cluster is associated with a pair of TS1 with lengths ranging from 88.0 to 117.0 µm and 1.33 to 1.68 µm in diameter, and a pair of shorter sensilla trichodea (TS2) with mean lengths of 56.82 ± 3.26 µm and 0.79 ± 0.07 µm in diameter.

Two types of stellate pores are present in the adult male of *P. solenopsis*; type SP2 has 5 projections (peripheral loculi) resembling short sensillum basiconicum (1.40 ± 0.08 µm in length and 0.43 ± 0.02 µm in diameter). The type SP3 has 6 projections (Fig. 3E). These stellate pores have an outside diameter of 4.20–5.03 µm and an inside diameter of 3.38–4.00 µm. In addition, the medial part of the last abdominal segment has microtrichia (Mt) which are arranged singly or in pairs; their lengths ranging from1.00 µm to 1.13 µm (Fig. 3F).

**Figure 4:**
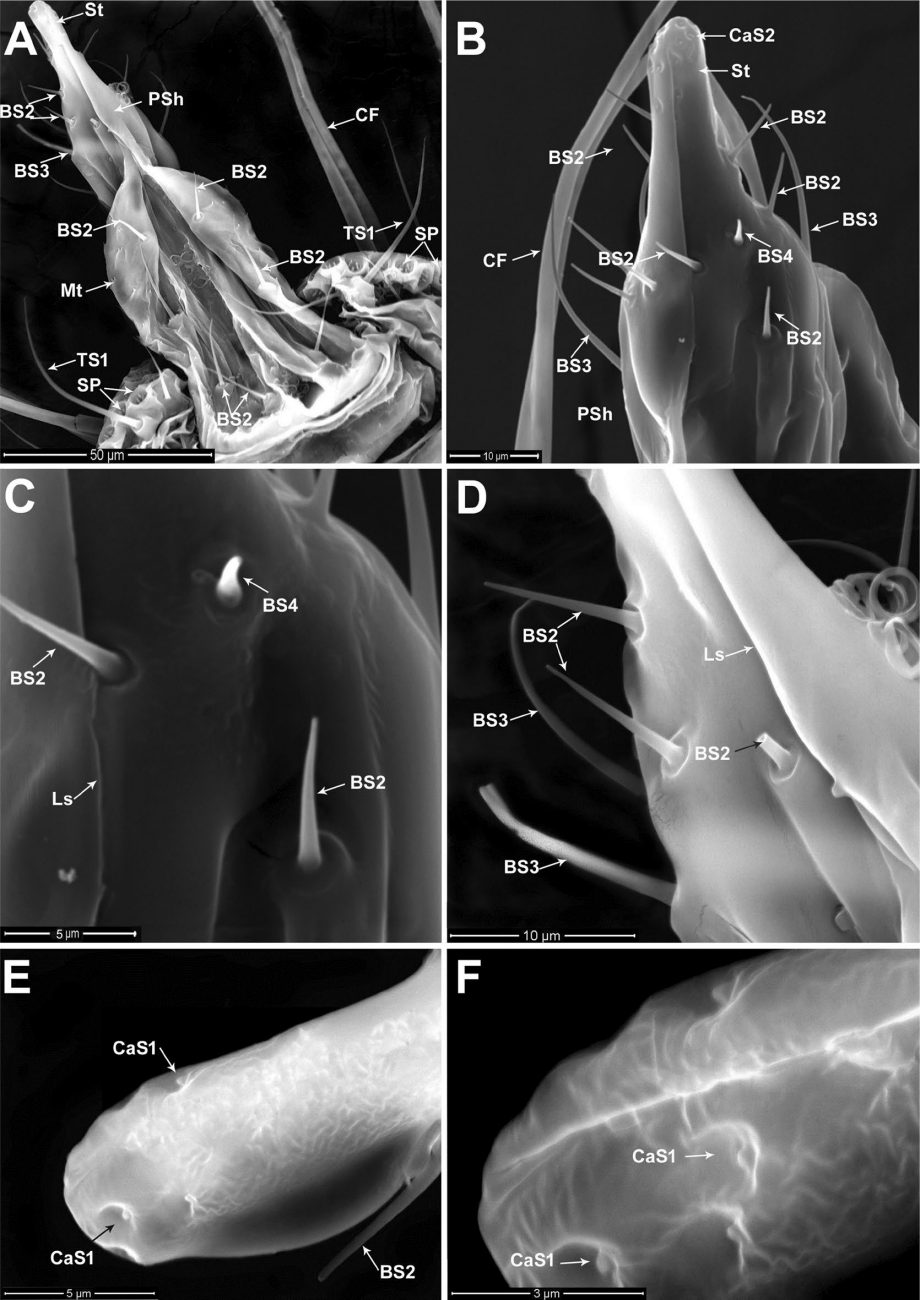
Genitalia of adult male of *P. solenopsis*: (**A**) Ventral view of the genital segments and a part of 8th abdominal segment with two pairs of caudal filaments (CF) surrounded by various groups of stellate pores (SP) and sensillum trichodeum (TS1). The style (St) illustrated at the distal end of the penile sheath (PSh) with various sensilla basiconica (BS2, BS3) and microtrichia (Mt). (**B**) Magnified ventral view of the terminal part of the last abdominal segment showing parts of caudal filaments (CF), the penile sheath (PSh) bearing various types of sensilla basiconica (BS2, BS3, BS4), and sensilla campaniformia (CaS1) at the tip of the style (St). (**C**) Magnified ventral view of the middle part of the penile sheath (PSh) showing sensilla basiconica (BS2), short sensillum basiconicum subtype 4 (BS4), and longitudinal slit (Ls) of the penile sheath. (**D**) Part of the penile sheath with longitudinal slit (Ls) showing magnified view of sensilla basiconica (BS2, BS3), broken basiconic sensillum subtype 2 (black arrow). (**E & F**) Magnified dorsal and ventral views of sensilla campaniformia (CaS1) on the terminal part of the style, respectively.

The dorsal surface of the ninth and tenth abdominal segments has a strongly sclerotized triangular arrangement; however, it appears largely membranous towards the ventral portion around the base of the aedeagus (Figs. 3A,B, 4A). Ventrally, the genital segments are modified as the incompletely fused ninth and tenth abdominal segments which compose the penile sheath that is characterized by a longitudinal slit along the median ventral part, and its distal part terminates in an evenly rounded tip called the style (Fig. 4A,B).

**Figure 5:**
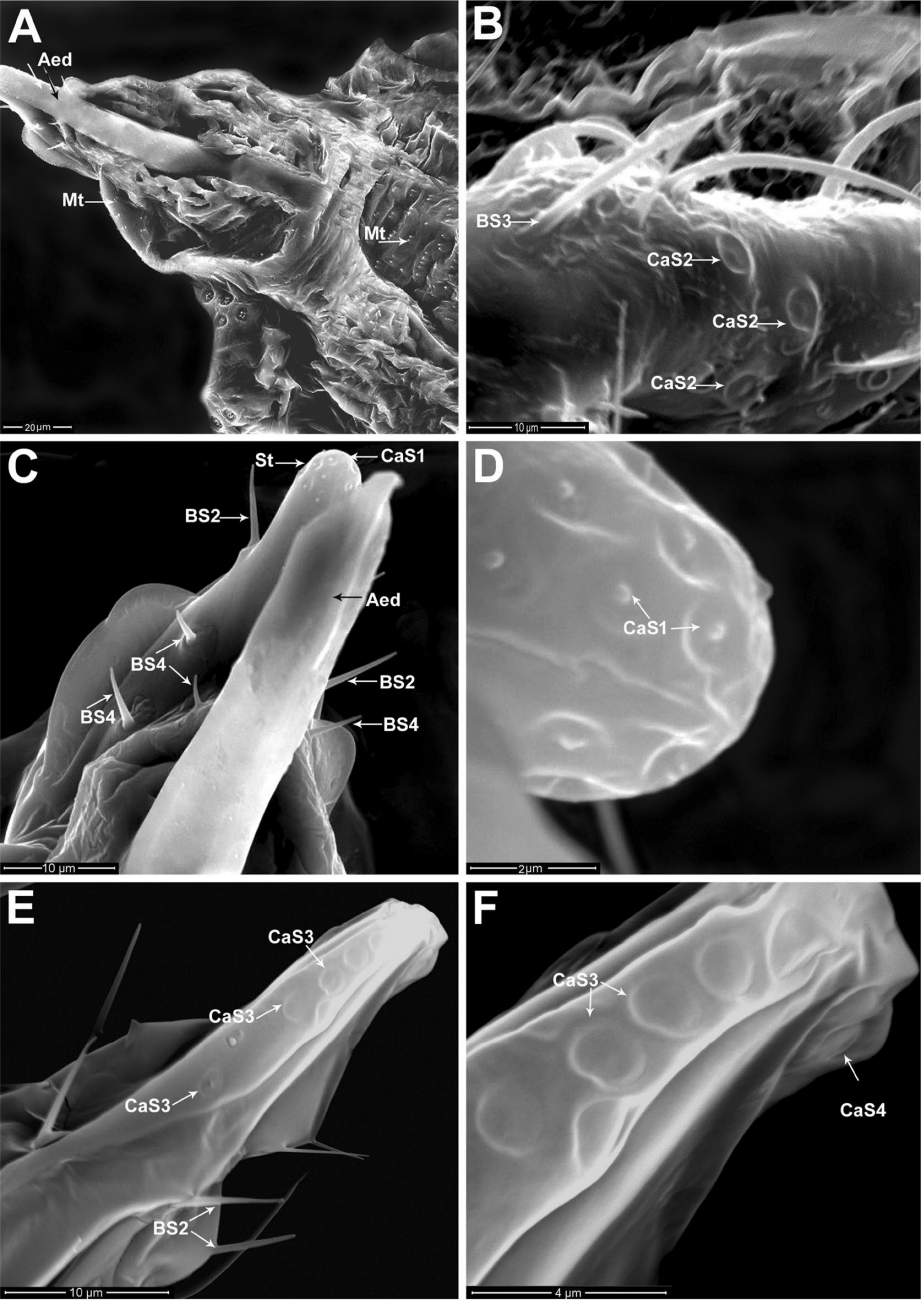
Genitalia of adult male of *P. solenopsis*: (**A**) Ventral view of the genital segments, showing extended aedeagus (Aed) and rows of microtrichia (Mt) located on the last abdominal segments. (**B**) Magnified lateral view of the middle abdominal segment with sensilla campaniformia subtype 2 (CaS2) and group of sensilla basiconica (BS3). (**C**) Magnified view of the terminal part of the genital segment showing the tip of aedeagus (Aed), style (St) associated with sensilla campaniformia subtype 1 (CaS1), and various sensillum types (BS2, BS4) on the penile sheath. (**D**) Magnified view of sensilla campaniformia subtype 1 (CaS1) on the distal part of the style. (**E**) Lateral view of the last abdominal segments shows sensilla campaniformia subtype 3 (CaS3) and sensilla basiconica (BS2). (**F**) Magnified view of sensilla campaniformia subtypes 3 and 4 (CaS3, CaS4).

Three subtypes of sensilla basiconica, BS2, BS3, BS4, were distributed on both sides of the penile sheath (Fig. 4C,D). Type BS2, which is the most abundant sensillum of the basiconica type, with a mean length of 7.91 ± 0.71 µm and 0.86 ± 0.04 µm basal diameter. BS3 is the longest sensilla, with a mean length of 22.96 ± 1.19 µm and 0.76 ± 0.08 µm in diameter. BS4 is the shortest subtype of the sensilla basiconica with a mean length of 4.37 ± 0.54 µm and 0.79 ± 0.07 µm in diameter. The terminal portion of the style has a group of sensilla campaniformia (CaS1) (Fig. 4E,F). The silt (sutural line) of the penile sheath allows the protrusion of the aedeagus during copulation, (Fig. 5A). Dorsally, the genital segments are characterized by a small, sclerotized area that represents the fused tergites of the ninth and tenth abdominal segments. The lateral surfaces of each genital segment have numerous BS3 and CaS2 (Fig. 5B).

The aedeagus is connected ventrally to the penile sheath wall behind the basal ridge (Fig. 5A). The aedeagus appears as a long, strongly cylindrical tube with curved, hook-shaped, trapped apex (Fig. 5C). Groups of CaS1 are associated with the tip of the penile sheath (Fig. 5D). At the dorso-distal margin of the penile sheath, there are two subtypes of sensillum campaniformium, namely, a group of CaS3 and a unique CaS4 (Fig. 5E,F).

## Discussion

The wax secretions produced by various hemipteran species, especially among mealybugs (Pseudococcidae), whiteflies (Aleyrodidae), aphids (Aphididae), scale insects (Coccidae), and flatid planthoppers (Flatidae), have been reported to cover all or part of their integuments^23^. The wax pores and ducts of the immature and adult stages of mealybug species, seen as trilocular pores and tubular duct glands, can synthesize waxy substances. The components for the waxy secretion are transferred from the haemolymph, stored in reservoirs, then secreted through the wax pore canals onto the surface of the integument^2^. The immature stages that are destined to develop as adult males construct a loosely woven, silky, filamentous cocoon after the second moult and undergo a further two moults as prepupal and pupal stages inside the cocoon before they emerge as winged adults^14,15,24,25^. The adult males are short-lived and non-feeding^21^.

Taxonomists use the types and distributions of the wax-producing pores and their ducts as an invaluable aid in the identification and classification of different mealybug species. For the family Pseudococcidae, several studies have focused on the female wax pores; however, few studies have been performed on the ultrastructure of the wax pores of the adult male^5,11^.

In that context, our study aimed to fill part of the knowledge gap on mealybug males and their nymphal instars. Results of the present study indicate that tubular ducts are more numerous in the second instar male; however, trilocular pores distributed among both nymphal stages. Trilocular pores are characterized by the production of a long, spiral wax filaments. However, the wax produced from the pores differs in the adult from that of the immature male instars^5,11^.

For the winged male, our morphological findings are very similar to those for the three species of mealybugs, *F. virgate*, *P. manihoti*, and *Pl. citri*^11^. The wax pores (pleural pores) of *M. hirsutus* are distinctively different in the adult males from those in the instars^5^. The function of the loose, filamentous wax on the body surface of the adult male was difficult to determine^5^. On the other hand, the pleural pores of the *Pl. citri* male which are merely vestiges from the immature stages and only found in adult that largely produces a white, powdery wax which trails behind the body could help with balance during the flight process^11^. Another report has also suggested that the pleural pores of the winged male of *M. hirsutus* are a probably a vestige from the immature stages^5^. Furthermore, the same authors described the morphological structures of the cluster of stellate pores containing four-five loculi which produce long spiral wax filaments^5,11^. Our findings regarding stellate pores morphology are very similar to^11^, except for the number of the loculi, which is five-six in the present study for *P. solenopsis* male. Therefore, the number of loculi of the stellate pores of the glandular pouch on the seventh and eighth abdominal segments should be characteristic for the differentiation between mealybug species.

For the nymphal instars of the cotton mealybug*,* the anal ring is associated with 3 pairs of long sensilla chaetica (ChS). Our findings match those reported for the adult females of *Pl. citri, Pl. ficus*, and *P. solenopsis*^26,27^. However, neither the ultrastructure description nor details on the sensillum types and their distribution are available for the prepupal stage of *P. solenopsis*, or any mealybug species. This lack of information has been addressed partially but significantly by the present study's findings, which suggest the value of these morphological characters for the discrimination of prepupal stages. Numerous tubular ducts and trilocular pores were distributed on the integument of the *P. solenopsis* nymphs. In addition, the prepupa has 3 pairs of long ChS, such as those found in the nymphal stages.

In adult mealybug males, the sensilla trichodea, basiconica, and campaniformia distributed on the surface of the genitalia may have an important role in mating events and copulatory behavior. Discerning from the morphological characteristics of the mentioned sensillum types, long and slender sensilla trichodea (ST1 and ST2) with a pointed tip are candidates for tactile mechanoreception, while some short sensilla basiconica (SB) with presumed terminal pore or wall pores could have an olfactory function^28^. Furthermore, the presence of numerous sensilla campaniformia on the terminal portion of the style of the penis of the cotton mealybug male is particularly interesting to consider. These sensilla are frequently in great numbers on the male genitalia of certain insects from different taxonomic groups, such as the uncus of the cabbage white butterfly, *Pieris brassicae* Linnaeus (Lepidoptera)^29^; the genital valves, epiproct and paraprocts of the Mediterranean katydid, *Phaneroptera nana* Fieber (Orthoptera)^30^; and the aedeagus of the false firefly beetle, *Drilus mauritanicus* Lucas (Coleoptera)^31^. In a hemipteran, the tip of the aedeagus in the soapberry bug, *Leptocoris augur* Fabricius, only bears numerous, short sensilla trichodea, sometimes misinterpreted as sensilla coeloconica, but do not bear sensilla campaniformia^32^. All the sensilla campaniformia of insects are mechanoreceptors and proprioceptors that respond to the stresses and strains in the exoskeleton of insects^33,34^, i.e., they monitor mechanical deformation of the cuticle. During the courtship behavior of mealybugs, these receptors may be having important role by strong touch of male genital stimulation, causing a response of the female^33,34^.

## Conclusions

This study explored the ultrastructure of wax pores of both the adult male and immature stages of the cotton mealybug*, P. solenopsis* Tinsley. In addition, the adult genital structures were imaged for the first time with a SEM. These findings provide useful insights that can help in the identification of the nymphal stages of *P. solenopsis* found on produce at pre-export inspections. Information generated for the adult male hopefully enriches our knowledge to better understand the morphology of the traits that contribute to the biology and behavior, especially mating events and copulatory behavior of *P. solenopsis*.

## Materials and methods

### Insects

A colony of the cotton mealybug, *P. solenopsis*, was established in the laboratory of Pests & Plant Protection Department, Agricultural & Biological Research Institute, National Research Centre, Giza, Egypt. The colony was reared on sprouting potato tubers (*Solanum* *tuberosum*). Studies on plants complied with relevant institutional, national, and international guidelines and legislation.

The mealybug colony was established under laboratory conditions (temperature of 26 ± 2 °C, 60–70% relative humidity, and 16 h light: 8 h dark photoperiod), in glass jars (30-cm deep and 15-cm width). Both immature stages and adult males were manually separated from the colony in order to prepare them for scanning electron microscopy (SEM) examination.

### Sample preparation for SEM

Fifteen samples of both immature stages and adult males were collected from the laboratory colony and stored in 70% ethanol, followed by a gradual dehydration using a series of ethanol concentrations (80%, 90%, 95% and 100% [v/v]), without distorting the samples. The waxy layer that affects the investigation process of such fine structures as sensilla was removed by soaking in hexane for 10 min, according to Abd El-Ghany et al.^12,35^. Samples of immature stages and adult males were rinsed in 100% ethanol, oriented and mounted on aluminum stubs with double sticky tape. The mounted samples were coated with gold film using the High Resolution Turbomolecular-pumped coater system (Q150T ES, Quorum Technologies Ltd, United Kingdom). The SEM Model TESCANVEGA3 (thermionic emission SEM system) (TescanTM, Tescan Orsay Holding, Kohoutovice, Czech Republic) was used to capture micrograph of the desired parts at high magnification ranging from 2500 to × 30,000.

### Nomenclature and morphological description of sensilla

The nomenclature used and identification of various sensillum types and wax pores was performed according to earlier literature^11,16–18^. The mean length and standard deviation were determined from measurements of 5 samples. The measurements were performed using ImageJ software (http://imagej.nih.gov/ij/).

## Data Availability

The datasets generated during and/or analyzed during the current study are available from the corresponding author on reasonable request.

## References

[1] Lambdin P, Capinera JL (2004). Scale insects and mealybugs (Hemiptera: Coccoidea). Encyclopedia of Entomology.

[2] Zhang Y, Xie Y, Xue J, Fu X, Liu W (2012). The structure of integument and wax glands of *Phenacoccus fraxinus* (Hemiptera: Coccoidea: Pseudococcidae). Zool. Res..

[3] Gullan PJ, Cranston PS (2005). The Insects: An Outline of Entomology.

[4] Cox JM (1987). Pseudococcidae (Insecta: Hemiptera). Fauna N. Z..

[5] Kumar V, Tewari SK, Datta RK (1997). Dermal pores and wax secretion in mealybug *Maconellicoccus hirsutus* (Hemiptera, Pseudococcidae), a pest of mulberry. Ital. J. Zool..

[6] Gullan PJ (2000). Identification of the immature instars of mealybugs (Hemiptera: Pseudococcidae) found on citrus in Australia. Aust. J. Entomol..

[7] Wakgari WM, Giliomee JH (2005). Description of adult and immature females of six mealybug species found on citrus in South Africa. Afr. Entomol..

[8] Hardy NB, Gullan PJ, Hodgson CJ (2008). A subfamily-level classification of mealybugs (Hemiptera: Pseudococcidae) based on integrated molecular and morphological data. Syst. Entomol..

[9] Sirisena UGAI, Watson GW, Hemachandra KS, Sage O, Wijayagunasekara HNP (2015). Scanning electron microscopy of six selected mealybug (Hemiptera: Pseudococcidae) species of Sri Lanka. Trop. Agric. Res..

[10] Rezeki MS (2021). Identification key to nymphal and adult mealybugs (Hemiptera: Pseudococcidae) associated with dragon fruits in Indonesia. Biodiversitas.

[11] Cox JM, Pearce MJ (1983). Wax produced by dermal pores in three species of mealybug (Homoptera: Pseudococcidae). Int. J. Insect Morphol. Embryol..

[12] Abd El-Ghany NM, Zhou JJ, Dewer Y (2022). Antennal sensory structures of *Phenacoccus solenopsis* (Hemiptera: Pseudococcidae). Front. Zool..

[13] Franco JC, Zada A, Mendel Z, Ishaaya I, Horowitz A (2009). Novel approaches for the management of mealybug pests. Biorational Control of Arthropod Pests.

[14] Vennila S (2010). Biology of the mealybug, *Phenacoccus solenopsis* on cotton in the laboratory. J. Insect Sci..

[15] Sileshi G (2019). Biology of cotton mealybug *Phenacoccus solenopsis* (Tinsley) on cotton plants under the laboratory conditions. Acad. Res. J. Agric. Sci. Res..

[16] McKenzie HL (1967). Mealybugs of California with Taxonomy, Biology and Control of North American Species (Homoptera: Coccoidea: Pseudococcidae).

[17] Afifi SA (1968). Morphology and taxonomy of the adult males of the families Pseudococcidae and Eriococcidae (Homoptera: Coccoidea). Bull. Brit. Mus. Entomol. Suppl..

[18] Kosztarab M, Kozár F (1988). Scale Insects of Central Europe.

[19] Duelli P (1985). A new functional interpretation of the visual system of male scale insects (Coccida, Homoptera). Experientia.

[20] Mendel Z (2012). Sexual maturation and aging of adult male mealybug (Hemiptera: Pseudococcidae). Bull. Entomol. Res..

[21] Silva EB, Mouco J, Antunes R, Mendel Z, Franco JC (2009). Mate location and sexual maturity of adult male mealybugs: Narrow window of opportunity in a short lifetime. Pheromones Semiochem. IOBC/wprs Bull..

[22] Miller DA, Rung A, Parikh G (2014). Scale insects, edition 2, a tool for the identification of potential pest scales at USA ports-of-entry (Hemiptera: Sternorrhyncha: Coccoidea). ZooKeys.

[23] Ammar E-D, Alessandro RT, Hall DG (2013). Ultrastructural and chemical studies on waxy secretions and wax-producing structures on the integument of the woolly oak aphid *Stegophylla brevirostris* Quednau (Hemiptera: Aphididae). J. Microsc. Ultrastruct..

[24] Dhawan AK (2007). Incidence and damage potential of mealy bug, *Phenacoccus solenopsis* Tinsley on cotton in Punjab. Indian J. Ecol..

[25] Prasad YG, Prabhakar M, Sreedevi G, Ramachandra R, Venkateswarlu B (2012). Effect of temperature on development, survival and reproduction of the mealybug, *Phenacoccus solenopsis* Tinsley (Hemiptera: Pseudococcidae) on cotton. Crop Prot..

[26] Nagrare VS (2011). Compendium of Cotton Mealy Bugs.

[27] Khalifa EA, El-Sebaey IIA, Zein HS, El-Deeb MM (2019). Taxonomic studies of common genera and species of family *Pseudococcidae* (Hemiptera: Coccoidea) with a taxonomic key for the species in Egypt. Egypt. J. Plant Prot. Res. Inst..

[28] Zacharuk RY, Kerkut GA, Gilbert LI (1985). Antennae and sensilla. Comprehensive Insect Physiology, Biochemistry and Pharmacology.

[29] Faucheux MJ (1999). Biodiversity and Unity of Sensory Organs in Lepidopteran Insects.

[30] Faucheux MJ (2012). The structure of the male postabdomen and associated sensilla of *Phaneroptera nana* Fieber 1853 and remarks on uniporous sensilla of genitalia (Orthoptera: Tettigoniidae: Phaneropterinae). Bull. Inst. Sci. Rabat Sect. Sci. Vie..

[31] Faucheux MJ, Beaulieu G, Agnas MB (2016). Deux espèces marocaines de Drilini (Coleoptera: Elateridae: Agrypninae): *Drilus mauritanicus* Lucas 1849 et *Malacogaster passerinii* Bassi 1833: Morphologie larvaire et imaginale, écologie générale. (Two Moroccan species of Drilini (Coleoptera: Elateridae: Agrypninae): *Drilus mauritanicus* Lucas 1849 and *Malacogaster passerinii* Bassi 1833: Larval and imaginal morphology, general ecology). Bull. Soc. Sci. Nat. Ouest. Fr..

[32] Badwaik VJ, Barsagade DD (2021). Microstructure characterization of male and female external genitalia of soapberry bug, *Leptocoris augur* (Hemiptera: Rhopalidae). J. Appl. Biol. Biotech..

[33] McIver SB (1975). Structure of cuticular mechanoreceptors of arthropods. Ann. Rev. Entomol..

[34] Abd El-Ghany NM, Faucheux MJ (2022). The mouthparts and sensilla of the adult tomato leafminer moth, *Tuta absoluta* (Meyrick, 1917) (Lepidoptera: Gelechiidae). Arthropod. Struct. Dev..

[35] Abd El-Ghany NM, Abd El-Aziz SE (2021). Morphology of antennae and mouthpart sensilla in *Lasioderma serricorne* (fabricius) (Coleoptera: Anobiidae). J. Stored Prod. Res..

